# eMouseAtlas, EMAGE, and the spatial dimension of the transcriptome

**DOI:** 10.1007/s00335-012-9407-1

**Published:** 2012-07-31

**Authors:** Chris Armit, Shanmugasundaram Venkataraman, Lorna Richardson, Peter Stevenson, Julie Moss, Liz Graham, Allyson Ross, Yiya Yang, Nicholas Burton, Jianguo Rao, Bill Hill, Dominic Rannie, Mike Wicks, Duncan Davidson, Richard Baldock

**Affiliations:** MRC Human Genetics Unit, Institute of Genetics and Molecular Medicine, University of Edinburgh, Western General Hospital, Crewe Road, Edinburgh, EH4 2XU Scotland, UK

## Abstract

eMouseAtlas (www.emouseatlas.org) is a comprehensive online resource to visualise mouse development and investigate gene expression in the mouse embryo. We have recently deployed a completely redesigned Mouse Anatomy Atlas website (www.emouseatlas.org/emap/ema) that allows users to view 3D embryo reconstructions, delineated anatomy, and high-resolution histological sections. A new feature of the website is the IIP3D web tool that allows a user to view arbitrary sections of 3D embryo reconstructions using a web browser. This feature provides interactive access to very high-volume 3D images via a tiled pan-and-zoom style interface and circumvents the need to download large image files for visualisation. eMouseAtlas additionally includes EMAGE (Edinburgh Mouse Atlas of Gene Expression) (www.emouseatlas.org/emage), a freely available, curated online database of in situ gene expression patterns, where gene expression domains extracted from raw data images are spatially mapped into atlas embryo models. In this way, EMAGE introduces a spatial dimension to transcriptome data and allows exploration of the spatial similarity between gene expression patterns. New features of the EMAGE interface allow complex queries to be built, and users can view and compare multiple gene expression patterns. EMAGE now includes mapping of 3D gene expression domains captured using the imaging technique optical projection tomography. 3D mapping uses WlzWarp, an open-source software tool developed by eMouseAtlas.

## eMouseAtlas: a spatial framework for the embryo

eMouseAtlas is a unique resource for research on mouse development and its relation to human disease (Christiansen et al. 2006; Richardson et al. 2010; Venkataraman et al. 2008). eMouseAtlas is a comprehensive online resource to visualise mouse development, identify anatomical structures, determine developmental stage, and investigate gene expression in the mouse embryo. The eMouseAtlas portal page (www.emouseatlas.org) allows access to the anatomy atlas and the EMAGE database of gene expression. The anatomy atlas provides definitive models of successive stages of post-implantation mouse embryo development. 3D models of Theiler-stage embryos can be viewed as movies or in any arbitrary section plane at tissue-level resolution, and can be used to explore the embryo structure in depth. At most stages of development the 3D models are linked to high-resolution images of H&E-stained histological sections from the same specimen and these can be used to show the cellular and subcellular structure at any location. Anatomy in eMAP is represented as a formal ontology that is the de facto standard for annotation of spatial patterns in the embryo, allowing consistent textual queries across different databases. Uniquely in the case of eMouseAtlas, this formal ontology is linked to delineated anatomical domains within the 3D embryo models.

Also accessible from the eMouseAtlas portal page is EMAGE (Edinburgh Mouse Atlas of Gene Expression) (www.emouseatlas.org/emage), a freely available, curated online database of *in situ* gene expression patterns in the developing mouse embryo. Gene expression domains extracted from raw data images are spatially mapped into atlas embryo models. The embryo models provide a framework for the EMAGE gene expression database so that analysis can move from anatomy to embryo space and back. By using this unique framework, EMAGE introduces a spatial dimension to transcriptome data and allows exploration of the spatial similarity between gene expression patterns. Spatial analysis is especially relevant during development when domains of gene expression do not necessarily correspond with named anatomical structures. In addition, EMAGE allows for conventional text-based queries using gene or anatomical terms. EMAGE recently has extended its spatial mapping abilities to include mapping of 3D gene expression domains captured using the imaging technique optical projection tomography (OPT); this has had a significant impact on our ability to map coexpression of genes. EMAGE entries also link to external resources such as GXD, GEISHA, PubMed, Ensembl, Allen Brain Atlas, BioGPS, and IKMC, and this allows EMAGE spatial transcriptome data to be integrated with data from other sources.

In producing a spatial framework, eMouseAtlas plays an international leading role in the development of an informatics infrastructure for the wider developmental biology community. The eMouseAtlas web resource (www.emouseatlas.org) is used as an educational tool to teach mammalian developmental biology and is also used worldwide as a research tool. Critical to the project’s core principles is that the models, maps, and ontologies we produce are open source and available to all members of the scientific research community. For this reason, eMouseAtlas continues to be a freely available, publicly accessible web resource.

## Anatomy atlas and visualisation

**Fig. 1:**
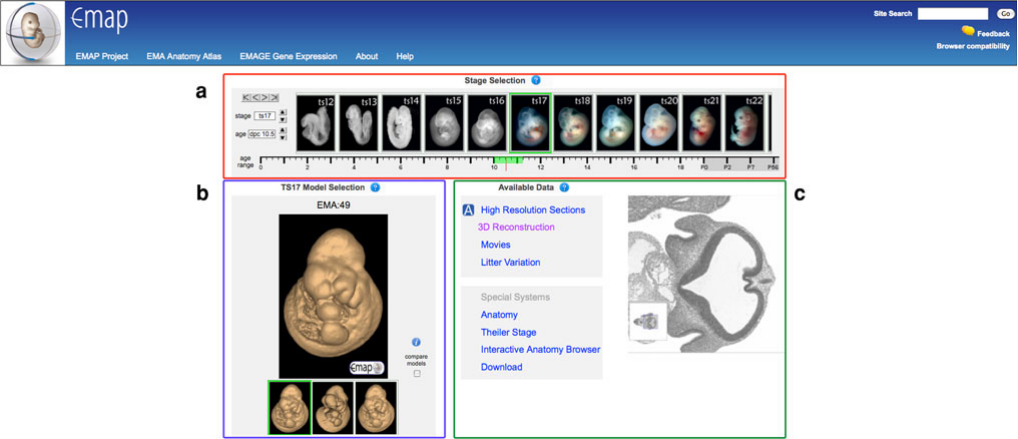
The eMouseAtlas main page. The “film-strip” embryo selector (**a**) can be scrolled via click and drag directly or by using the control buttons at the top left. Selecting a single model here influences what is displayed in the bottom half of the page. Each Theiler stage may contain multiple examples of data (usually littermates) and these can be individually selected here (**b**). Each embryo potentially can be viewed in a number of ways: the original high-resolution sections; a 3D reconstruction from the former; movies of the embryo and its littermates; associated information such as its anatomy ontology, Theiler staging, a section browser, and download options of the embryo data (**c**)

**Fig. 2:**
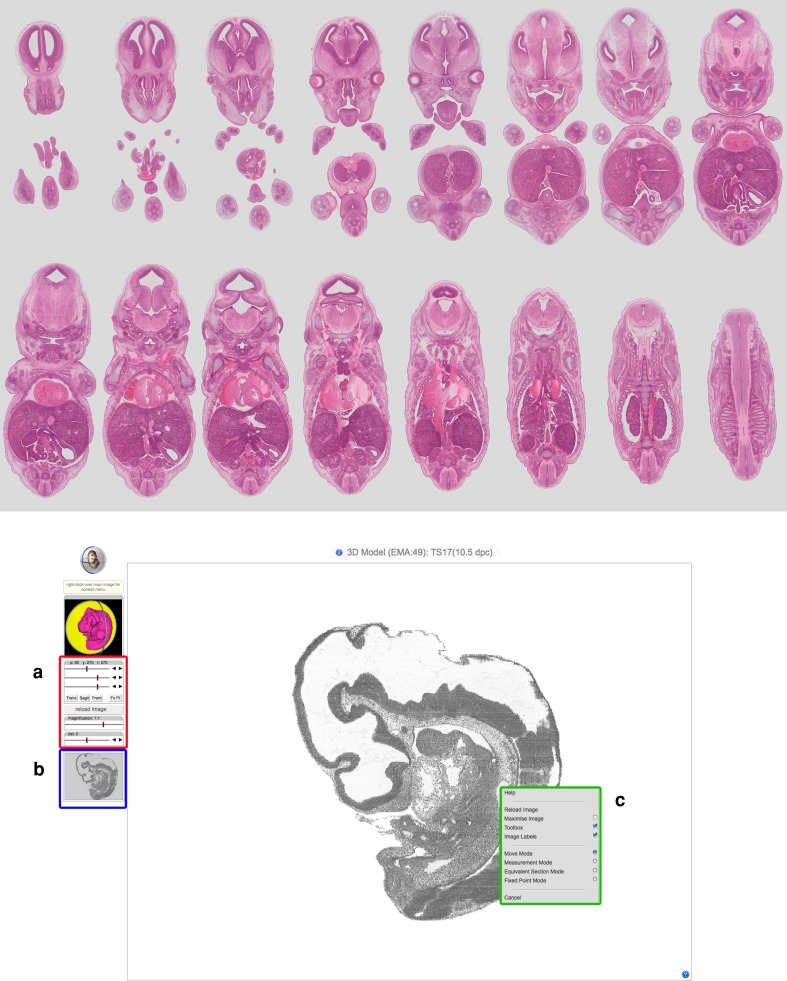
High-resolution sections and the IIP3D viewer. *Top* An example of the original sections that are digitised and could be used for creating 3D reconstructions. These are E14.5 (TS23, model EMA:83) H&E-stained high-resolution coronal histological sections. *Bottom* Typical IIP3D viewer window when viewing the “3D reconstruction” option from the main-stage view window on the EMA website. The IIP viewer window comprises a main viewer that is controlled by slider controls for pitch (p), yaw (y), roll (r), magnification, and distance (**a**). A guide to the plane being selected is provided in the view directly above. A real-time preview of the plane is provided in **b**, where the transparent grey box represents the viewable dimensions in the main viewer. Right-clicking (ctrl-click on Mac) in the main viewer brings up a context menu that allows, amongst other things, a measurement mode by clicking two arbitrary points in the main viewer in addition to being able to retrieve the original high-resolution image (see *top panel*) used to create the 3D reconstruction (**c**)

We have recently (March 2011) deployed a completely redesigned Mouse Anatomy Atlas website (www.emouseatlas.org/emap/ema) that uses a filmstrip to navigate between Theiler stages (Fig. 1). Theiler staging is based on morphological development of the mouse embryo, with embryogenesis divided into 26 Theiler stages (Theiler 1972). For each Theiler stage, a range of anatomy atlas models is available, including very-high-resolution histological section models, OPT, and HREM (high-resolution episcopic microscopy) reconstructions (Table 1). An IIP3D web tool (Husz et al. 2012) allows a user to view arbitrary sections of an eMAP model using a web browser (Fig. 2). This viewer is accessible by clicking on the “3D reconstruction” link for the appropriate Theiler stage. The displayed image, which may be only a small part of the overall image, consists of rectangular tiles served by a WlzIIPServer. Manipulations that can be performed on the displayed image include panning across the image, changing the image resolution, translating the viewing plane (i.e., distance), and rotating the viewing plane in three dimensions (pitch, yaw). Shortcuts are available to directly navigate to transverse, sagittal, and frontal views. In addition, there is the option to select layers, to toggle the visibility of layers, and to change the colour of layers. The latter option is particularly useful for visualising segmented anatomical domains that have been annotated in several eMAP models. Other features of the IIP3D viewer include the ability to measure the distance between two points on the embryo atlas models and to find the original high-resolution histological section used to generate the model.

**Table 1:** Database statistics

Total no. of models	63
Stages	TS07-26
Histology section reconstructions	20
With painted anatomy	12 (with 2 more pending)
OPT models	41
High-resolution episcopic microscopy	2
No. of reference models	17
MRI models	
Caltech μMRI	4
Total EMAGE entries (as of 03/2012)	40,770
Genes	16,014
EMAGE entries by source	
EurExpress (www.eurexpress.org)	16,872
EmbryoExpress (www.embryoexpress.org)	1,834
FaceBase (www.emouseatlas.org/emage/facebase/)	287
MGI (www.informatics.jax.org/) + direct submissions	21,777
Curated by EMAGE editors	23,145
EurExpress (www.eurexpress.org)	14,985
Voxel size of eMouseAtlas embryo models (μm)	
1 × 1 × 1	1 model (TS12)
2 × 2 × 2	6 models (TS07-12)
3 × 3 × 3	2 models (TS23)
4 × 4 × 7	8 models (TS13-17;19-20)
4.52 × 4.52 × 7	1 model (TS23)
4.53 × 4.53 × 5.6	1 model (TS18)
6.2 × 6.2 × 6.2	5 models (TS15)
7.8 × 7.8 × 7.8	3 models (TS16)
8 × 8 × 8	3 models (TS15;17)
9.9 × 9.9 × 9.9	5 models (TS18)
11 × 11 × 11	7 models (TS19)
13.3 × 13.3 × 14	1 model (TS26)
16.5 × 16.5 × 16.5	1 model (TS21)
17.5 × 17.5 × 17.5	8 models (TS23)
18 × 18 × 18	1 model (TS22)
19 × 19 × 19	4 models (TS24)
25 × 25 × 25	2 models (TS25)

In addition, the atlas website hosts an interactive anatomy browser that allows a user to access staged anatomy in an ontology tree. The eMAP ontology uses a controlled vocabulary and *part-of* relationships to describe anatomical components, with stage-specific partonomic (part-of) hierarchies provided from TS01-TS26. The recently developed interactive ontology viewer illustrates the partonomic relationships between the anatomical terms as a hierarchy where each term may have a number of subcomponents. Additional options allow users to query EMAGE, GXD, Google, and Wikipedia by selecting a component in the tree. The full ontology or stage-specific subsets are available for download in plain text, rtf, and xml formats. Furthermore, the “abstract” anatomy, or eMAPA ontology, is accessible through the interactive viewer and is available for download in the same formats plus the Open Biomedical Ontologies (obo) format. The eMAPA ontology is used as a reference index for mouse embryo data in both eMAP and EMAGE and is also used by external databases, including the GXD and the cross-species ontology resource Uberon. A preliminary version of the eMAPA ontology has been submitted to the National Center for Biomedical Ontology (NCBO). The adult mouse anatomical dictionary (AMA), complementary to the eMAP ontology, is maintained by the Jackson Laboratory.

## New features of the mouse atlas EMAGE resource

**Fig. 3:**
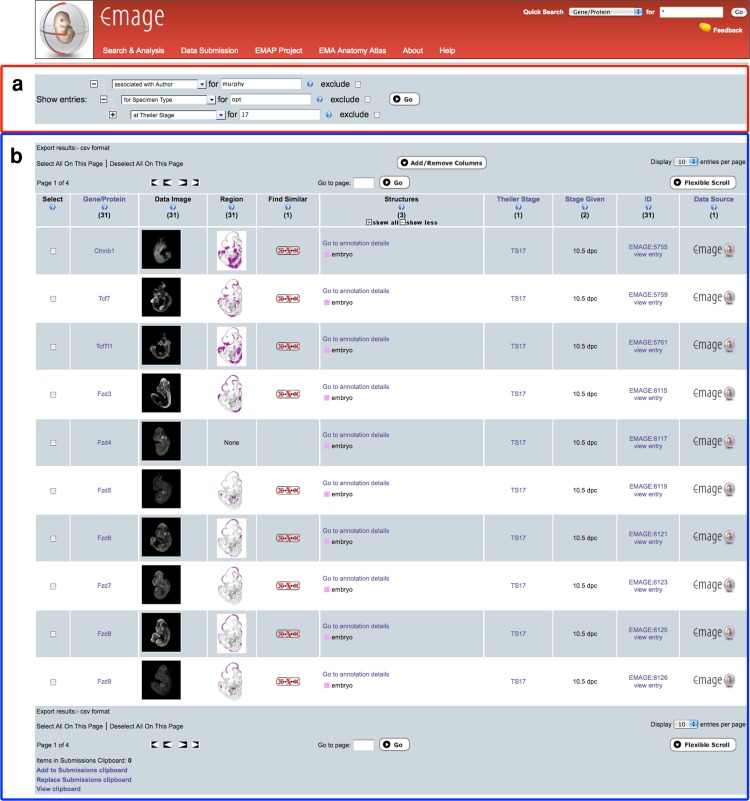
The EMAGE combination query feature allows a user to build complex queries. In this example, the combination query is used to find all OPT data with author Dr. Paula Murphy, Trinity College, Dublin, at a single stage of embryonic development. **a** An expandable multi-input selector allows a user to select criteria for a complex query. In this example, these include associated with author = murphy; specimen type = opt; Theiler stage = 17. Additional options include **b** A list of 31 OPT entries with author Dr. Paula Murphy at Theiler stage 17 is returned from the combination query. This list can be further refined by use of the expandable multi-input selector

**Fig. 4:**
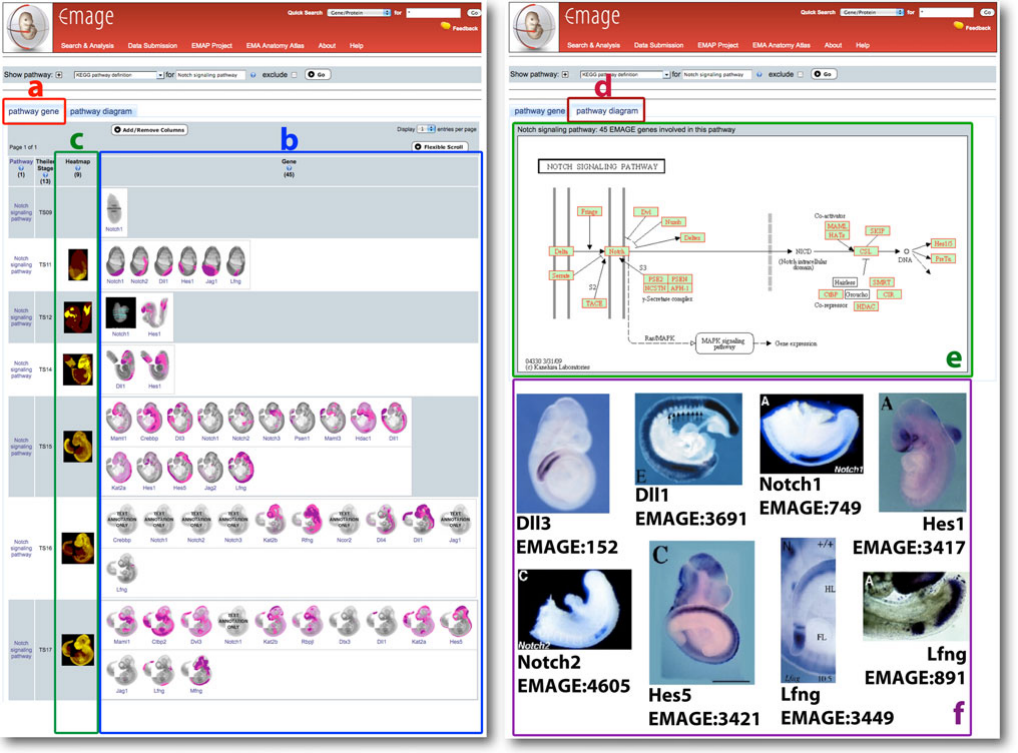
EMAGE Pathways uses KEGG to find gene expression patterns associated with specific pathways and/or specific tissues. In this example, EMAGE Pathways is used to find components of the Notch signalling pathway that are important in somitogenesis. Two tabs are returned from a pathway query. The first tab (**a**) is entitled “pathway gene” and shows gene expression summaries associated with all gene components of the KEGG Notch signalling pathway. Spatially mapped EMAGE data are shown in the Gene column; if spatially mapped data are unavailable, a thumbnail will display “text annotation only” (**b**). The Heatmap column shows the union of all spatially mapped expression patterns for a single KEGG pathway at a single stage (**c**). Clicking on the second tab (**d**) allows access to the KEGG pathway diagram (**e**). The KEGG pathway diagram is marked up to show data available in EMAGE for a given gene by highlighting these genes in red. A subset of Notch signalling pathway genes can be implicated in somitogenesis by virtue of their gene expression patterns (**f**). These include the Notch ligands *Delta-like 3* (EMAGE:152) and *Delta-like 1* (EMAGE:3691); the *Notch1* receptor (EMAGE:749); and the hairy-like gene *Hes1* (EMAGE:3417), all of which are expressed in presomitic mesoderm. In contrast, *Notch2* (EMAGE:4605) and *Hes5* (EMAGE:3421) are not expressed in the presomitic mesoderm but are expressed in the somites, suggesting that these are downstream effector genes in somite formation. In this respect it is additionally noteworthy that Delta-like 1 (EMAGE:3691) shows higher levels of expression in presomitic mesoderm than in the somites. *Lunatic Fringe* (EMAGE:3449; EMAGE:891) is expressed in discrete domains in the presomitic mesoderm and the condensing somite and illustrates the critical importance of this gene in forming boundaries between somites by a mechanism involving oscillatory inhibition of the Notch receptor

EMAGE is an extension of the atlas resource that has been used to archive and spatially map ~21,000 gene expression patterns from a variety of sources, including large in situ hybridisation screens (EurExpress, FaceBase), the GXD, and the published literature as well as unpublished direct submissions from developmental biology labs. Gene, anatomy, and embryo space queries that allow the user to find gene expression patterns have been described previously (Christiansen et al. 2006; Richardson et al. 2010; Venkataraman et al. 2008). To enable users to browse the EMAGE data more effectively, we have developed a Combination Query that allows complex queries to be built, and Gene and Pathway Summaries that allow users to view and compare multiple gene expression patterns using a web browser. The Combination Query includes gene, anatomy, ID, stage, GO term, and assay type and allows a user to construct a complex query step-by-step with additional options to include or exclude specific query terms (Fig. 3). The Gene Summary feature lists all the source images and spatial annotations for a given gene, at each Theiler stage, in a single “gene strip” that describes multiple EMAGE entries. The image summary tab of the gene strip allows users to view a montage of original images associated with a single gene and to identify details relating to specific images (e.g., authors, original publication, specimen details) easily using an intuitive browse-and-click interface. The Pathway feature organizes these gene strips using an outside resource, the Kyoto Encyclopedia of Genes and Genomes (KEGG), and uses KEGG Pathway maps to order gene strips according to their involvement in key signalling pathways (Fig. 4). The latter feature uses web services to query the KEGG resource and to integrate this query with the EMAGE database. Furthermore, we have recently deployed a Gene Association feature that uses a Gene and Pathway Summaries interface to deliver queries based on pathway, anatomical descriptions, and coexpression. This feature allows queries to be constructed using text-based descriptions (pathway, anatomy) and spatial information (coexpression).

### Full 3D embryo mapping

**Fig. 5:**
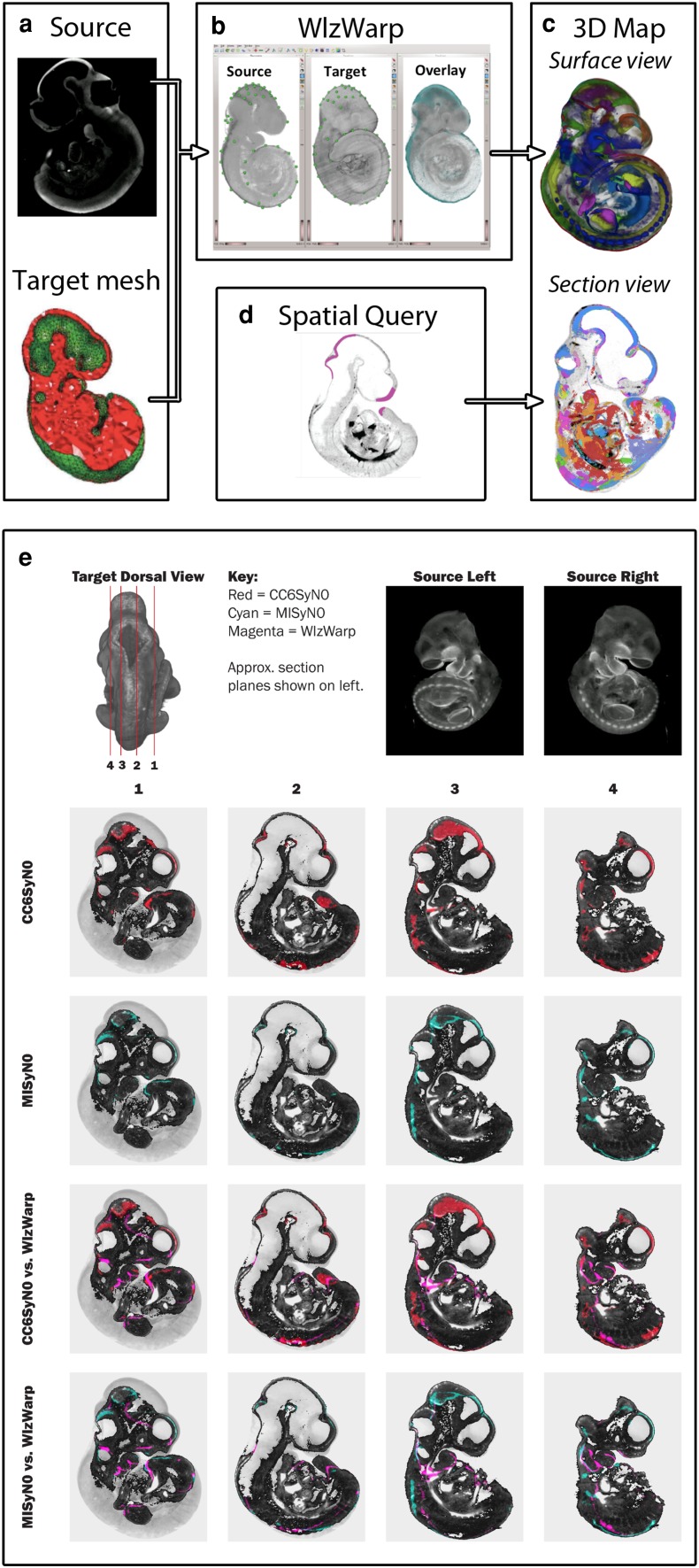
3D spatial mapping using WlzWarp. **a** 3D volume data are warped onto target reference models via a mesh that envelopes the target and excludes background and luminal areas. **b** The mesh is read into the WlzWarp interface, alongside the reference and source objects. WlzWarp allows user-defined pairwise landmarks to be placed on source and target and subsequently allows warping via the CDT algorithm. **c** 3D mapped data from multiple embryos can be viewed as a volumetric reconstruction or can be visualized on an arbitrary section by using a novel 3D search interface based on the IIP3D viewer. **d** Volumetric data can be queried in web browsers using a painted domain as the basis of a spatial query. **e** It is possible to refine 3D spatial mapping using advanced neuroimaging tools (ANTs) (Avants et al. 2011). These additional steps are performed after initial registration using WlzWarp. In this example, both cross-correlation (CC) and mutual information (MI)—two similarity metrics used to enhance 3D image registration—are used to refine the WlzWarp transformation of an OPT gene expression pattern. The target is the EMAP TS17 reference model, shown from its dorsal view (*top left*) with corresponding approximate section planes shown. The source embryo to be warped (*top right*) is an OPT gene expression pattern of *Wnt6* expression at the equivalent Theiler stage. ANTs can use various algorithms to perform automated nonrigid image registration. In the examples shown, CC and MI methods were tested subsequent to a manual 3D warping step using WlzWarp. The results are presented as a colour overlay of warped source (see key) onto a grey-scale section plane of the target. ANTs improves the overall registration of the source (compare CC6SyN0 vs. WlzWarp and MISyN0 vs. WlzWarp)

3D mapping is a powerful tool for the archiving, visualisation, and analysis of biological data. In some research fields, such as brain imaging, this has been achieved by adopting a stereotaxic coordinate system based on a small number of anatomical landmarks. However, this is not easily applicable in developmental biology, where the complex and changing morphology of the developing embryo precludes such a simple registration process. Embryo material is particularly variable in visual presentation and pose, i.e., the embryo can curl in a right-handed or left-handed spiral and the limbs can articulate. Consequently, spatial mapping requires extreme deformations of 3D volumes from a source image to a target reference. To address this issue, eMouseAtlas has developed an application that uses a constrained distance transform (CDT) (Hill and Baldock 2006) to deliver interactive 3D warping. An open-source cross-platform WlzWarp tool has been implemented; it is in routine use within EMAGE and is freely available to the community. WlzWarp has been successfully used to spatially map 3D gene expression patterns, obtained by imaging with OPT, to standardised models of embryonic development (Fig. 5). Mapped 3D expression patterns can be explored on arbitrary sections using the IIP3D viewer, and there is the additional option to find similar patterns of gene expression using a local spatial similarity search tool (LOSSST) algorithm. Our future plans are to develop this process to include automated 3D image registration procedures that will permit high-throughput 3D mapping of gene expression patterns with reduced interuser variability.

### BioMart

EMAGE has recently implemented a BioMart interface (http://biomart.emouseatlas.org) as a means of extending its range of queries (Stevenson et al. 2011). BioMart provides a generic web query interface and programmable access using web services. The interface is structured into several data sets providing the user with comprehensive query access to the EMAGE data, and allows for a greater range of possible queries that can include experiment details and other ancillary data. An important aspect of BioMart is that it allows federated queries between databases and could be used to invoke comparisons between EMAGE and other gene expression databases with a BioMart resource (e.g., GUDMAP, EurExpress, eChickAtlas). Importantly, BioMart cannot currently provide spatial searches or direct access to image data. The EMAGE web site and the BioMart interface therefore should be seen as complementary methods of querying EMAGE data content. We are currently exploring the feasibility of extending BioMart to spatially mapped data as this opens up the possibility of including spatial queries and filters and of integrating existing EMAGE BioMart queries with spatially organised data.

## Spatial analysis

eMouseAtlas is embracing the nascent research field of atlas informatics (Baldock and Burger 2012; Diez-Roux et al. 2011; Harding et al. 2011) by building on the existing resource to deliver novel analysis, systems biology, and data-mining methodology using the spatial context provided by atlas-based resources. An example of a novel analytical tool that has been developed is the paint-and-search Embryo Space feature accessible from the EMAGE home page that allows a user to paint a region on an image of a whole-mount embryo and use this painted domain to query the EMAGE database. This query tool returns a ranked list of spatially mapped gene expression patterns ordered by spatial similarity to the query region using the LOSSST (local spatial similarity search tool) algorithm. For any mapped EMAGE entry, a modified version of this algorithm is used in the “Find Similar Expression Patterns” feature. An example of a data-mining methodology that has been implemented at eMouseAtlas, and is made freely available, is the “Similar Patterns” tool that can be accessed from the EMAGE home page. This tool uses clustering of spatially mapped gene-expression patterns as a means of identifying in situ images with similar patterns. Clustering in this way provides evidence for potential interactions, which can be tested by other annotations (GO, KEGG, phenotype) and validated experimentally. This type of analysis extends to the complementary clustering based on anatomical annotation of gene-expression patterns (Diez-Roux et al. 2011), which also enables enrichment analysis to explore potential functional association.

We further plan to provide 3D spatial analysis of mapped OPT gene-expression patterns. The development of a standard coordinate space and the ability to map gene expression into this space will allow for 3D spatial analysis in the context of mouse development. In a pilot study, we are collaborating with Dr. Paula Murphy (Trinity College, Dublin) in investigating the spatial occupancy of *Wnt* signalling pathway OPT gene-expression domains in the developing mouse embryo. This study uses WlzWarp to map OPT gene-expression patterns from 19 mouse *Wnt* ligands and 10 *Frizzled* receptors at key stages of mouse development (Theiler stages 15, 17, and 19), and uses this set of 3D maps to provide spatial descriptions of gene enrichment across developmental time. It is anticipated that this work will help us to pinpoint and deliver a spatial analysis toolkit that can be used to establish syn-expression in a 3D context, with the potential of tracking these patterns through developmental time (4D).

## Current developments

### eMouseAtlas as a community resource for data sharing

eMouseAtlas has been a major MRC-funded programme for 16 years and is entering its fourth phase. Phase 1 developed the key atlas design, spatiotemporal database functionality, and basic image-processing tools. Phase 2 introduced an Editorial Office (EO) and converted the prototype to a functioning system with selected data. Phase 3 lifted the database from a relatively small system to a major international resource, unique in scope and content and embedded as a key resource for development and molecular genetics research. In this fourth phase, we propose to extend the database and web browser interfaces to make sure the whole research community can contribute to the eMouseAtlas as an integrating framework. Uniquely, we have begun to add atlas material from other sources as a step to widen community involvement in the production of a definitive resource. Thus far, additional atlas models include whole embryo (Dr T. Mohun, NIMR); forelimb and hind-limb models (Delaurier et al. 2008); kidney (EuReGene); Caltech MRI whole-embryo delineated models (Dr. S. Ruffins), and five to six somite embryos cultured for 24 h (Cajal et al. 2012). Furthermore, we propose to offer a portal for data sharing amongst the developmental biology community. The short-term goal for this project is to deliver a practical *community resource* that provides sharing and publication of 2D/3D embryo images. Whereas existing social networking services such as Facebook allow open sharing of 2D images, there is no facility in place to share, view, and publish 3D images using the World Wide Web, and none that can provide the scientific annotation and metadata tracking. eMouseAtlas provides interactive access to very-high-volume 3D images via a tiled pan-and-zoom-style interface, similar to the approach utilised by GoogleMaps. In addition to being very efficient, WlzIIP3D can be embedded in a standard tiled-image server to provide dynamic tile generation directly from virtual sections cut through an image volume. We have already demonstrated this technology for very large single images (up to 150 Gb), but this will extend to the multi-Tb images now being generated in biomedical research. We wish to extend the use of IIP3D technology by allowing researchers to upload, share, view, and publish 3D images using an interactive web-based application. The initial implementation will allow user data upload, sharing, and publication of 3D data. Critical in developing this interactive web-based app is the sharing of views and work in progress under the control of the user. The server technology that has been developed at eMouseAtlas, in particular, the ability to perform online queries of a high-volume 3D image archive, renders the eMouseAtlas resource perfectly suited as a long-term repository for large-scale 3D biological images such as those provided by OPT and MRI. Many research projects have a condition of funding that data are made publicly available at the end of the project and it is anticipated that the eMAP repository will provide a structured and managed mechanism for researchers to satisfy this requirement.

### eMouseAtlas and Kaufman’s atlas of mouse development

**Fig. 6:**
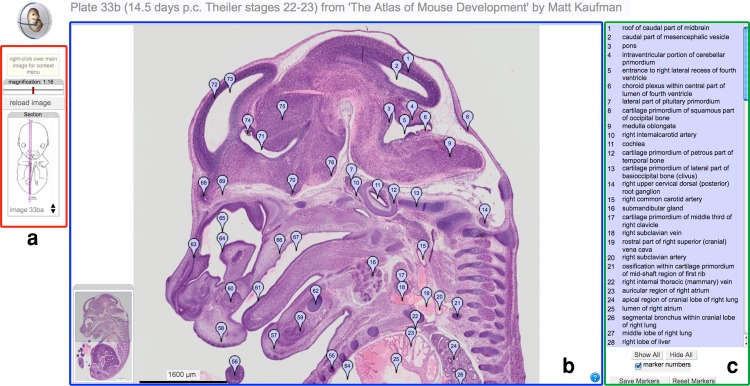
The interactive Kaufman’s atlas of mouse development. **a** Distance and magnification sliders control the main IIP viewer window display; the distance slider allows navigation through plates that appear in the original book. **b** The original sections have been digitised to generate high-resolution colour images that correspond to the plates used in the book. **c** These plates are annotated using Kaufman’s original terms plus the corresponding eMAP ontology terms, enabling direct links to eMouseAtlas and the EMAGE gene expression database. Anatomical components are flagged, and mousing over a flag highlights the corresponding text term and vice versa

eMouseAtlas is negotiating a licensing agreement from the publishing house Elsevier to deliver an online version of plates from *The Atlas of Mouse Development* by Matt Kaufman (1992). In collaboration with Elsevier, we are developing an online interface that uses the IIP3D server to deliver high-resolution colour images of the original histological sections used in Kaufman’s atlas (Fig. 6). The original sections have been digitised to generate >1,000 images that correspond to the plates used in the atlas. These plates are annotated using Kaufman’s original terms plus the corresponding eMAP ontology terms enabling direct links to eMouseAtlas and the exploration of partonomic relationships, gene-expression patterns, and “wiki”-based definitions and descriptions associated with each term.

### eMouseAtlas and spatial transcriptomics

EMAGE has comprehensive transcriptome data in the form of sparse section series. We have developed a trained colour-segmentation model to separate signal (in situ hybridization) from histology and background in the EurExpress data set, and we map this data in two ways. The first is a rapid-pass pseudo-whole-mount approach to provide an overview pattern for query and analysis. This is already in use in EMAGE and about 15,000 patterns from serial section data (EurExpress) have been spatially mapped. The extended approach maps this sparse section data into the full 3D space of the model embryo and interpolate the data to enable 3D query and analysis. This combined with the automatic signal extraction provides an automatic process to map the data in 3D. This is currently being tested on the EurExpress and EmbryoExpress data sets (19,000 transcripts).

In addition, we plan to apply a transcriptomic tomography technique to the embryo to deliver a low-resolution but complete spatial transcriptome using next-generation sequencing technologies. Drs. Okamura-Oho and Yokota of the RIKEN Wako Institute have developed a tomographic technique to capture the full-expression map of the murine brain (Okamura-Oho et al. unpublished). The technique is based on tissue sectioning with block-face imaging to allow reconstruction in conjunction with tissue collection and expression analysis (using standard microarray chips). In collaboration with Drs. Okamura-Oho and Yokota, we propose to pilot the technique for whole-mouse embryos. We will use Next Gen RNA-Seq to capture the full transcriptome of the 14.5-dpc embryo and to enable cross-validation of expression profiles with EurExpress in situ hybridisation data. To generate a 3D map, embryos will be serially sectioned in each of three orthogonal planes: frontal, sagittal, and transverse. If this strategy is successful, then we will develop a larger-scale proposal to apply this technique across a full range of stages from 9.5 to 17.5 dpc to deliver low-resolution but transcriptionally complete maps of the mouse embryo.

In addition to delivering the complete spatial transcriptome at key stages of mouse development, we propose to develop online tools that would allow analysis of spatial data and to compare this with more conventional text-based annotation. It is noteworthy that a tool for comparative analysis is already in place as part of the Argudas project (McLeod et al. 2012). The Argudas tool evaluates in situ gene-expression patterns by comparing text annotation associated with EMAGE and GXD entries. An extension of this tool that evaluated spatial annotation would be useful in delivering comparisons between spatial data and text-based annotation.
